# Infant Spinal Anesthesia for Urologic Surgery in a Patient With Epidermolysis Bullosa

**DOI:** 10.1155/cria/2537488

**Published:** 2025-07-01

**Authors:** Alexander B. Froyshteter, Jillian A. Dibiase, Catherine M. Seager, Alina Lazar

**Affiliations:** ^1^Department of Anesthesiology, Ann & Robert H. Lurie Children's Hospital of Chicago, 225 East Chicago Avenue, Chicago, Illinois 60611, USA; ^2^Department of Urology, Ann & Robert H. Lurie Children's Hospital of Chicago, 225 East Chicago Avenue, Chicago, Illinois 60611, USA

**Keywords:** epidermolysis bullosa, infant, spinal anesthesia

## Abstract

Infant spinal anesthesia presents a viable alternative to general anesthesia for short procedures below the umbilicus. This technique eliminates the need for airway instrumentation while preserving hemodynamic and respiratory parameters. Epidermolysis bullosa is a rare inherited genetic disorder marked by mucocutaneous fragility, erosions, ulcerations, and blister formation. Anesthesia management for patients with epidermolysis bullosa necessitates meticulous planning to minimize shearing stress on the skin and mucosa. This case report details the application of spinal anesthesia for an infant with epidermolysis bullosa for urologic surgery, highlighting special considerations for neuraxial block placement in this vulnerable population.

## 1. Introduction

Epidermolysis bullosa (EB) is an inherited genetic disorder characterized by mucocutaneous fragility, leading to erosions, ulcerations, and blister formation [1].

Its incidence is approximately 19.6 per one million live births [2]. This mechanobullous disorder comprises several subtypes categorized into four main groups based on the location of skin plane disruption and molecular protein abnormalities [2]. The severity of symptoms varies within subgroups and depends on the layer of disruption. EB simplex, the most common subtype, accounts for about 70% of cases and is characterized by erosions and blisters that heal without scarring, although hyperpigmentation may occur [3]. Since the oral mucosa is composed of stratified squamous epithelium, its integrity is compromised by dysfunction of certain proteins, leading to varying degrees of mucosal blistering across subtypes [3]. In more severe subtypes, laryngeal and tracheal lesions can form which may lead to glottic and tracheal scarring and subsequent stenosis [3].

Anesthesia for patients with EB requires careful planning to mitigate shearing stress on the skin. Attention should also be given to the types of dressings and lubricants used for skin care management [4–6]. Monitor application and intravenous line securement are typically done without the use of adhesives and should follow hospital-specific protocols that utilize products approved for EB patients [6].

General anesthesia is commonly used for EB patients but poses a significant risk of blistering during airway manipulation [5]. Sedation alone is often insufficient and regional blocks may provide inadequate surgical analgesia. Recently, infant spinal anesthesia (SA) has emerged as a safe alternative to general anesthesia for short infraumbilical procedures [7–9]. SA avoids airway manipulation, reducing the risk of airway and respiratory complications, and is performed with minimal trauma to the tissues. Despite its advantages, SA has been reported in limited cases involving children with EB [10, 11]. Reluctance to adopt this technique may stem from concerns about potential tissue injury while holding the infant in the typical sitting position for spinal block placement, the shorter duration of the block compared to adults, and the risk of needing to convert to general anesthesia or manage the airway in less-than-ideal conditions. Additionally, limited experience with this technique contributes to its cautious application. However, given recent advancements in management strategies, the use of SA in EB patients warrants further exploration. This case report highlights the feasibility of using SA for combined inguinal hernia repair and circumcision in an EB patient, focusing on key considerations for success.

The patient's family have provided written Health Insurance Portability and Accountability Act (HIPAA) authorization consent to publish this case report and associated patient figures.

## 2. Report

**Figure 1:**
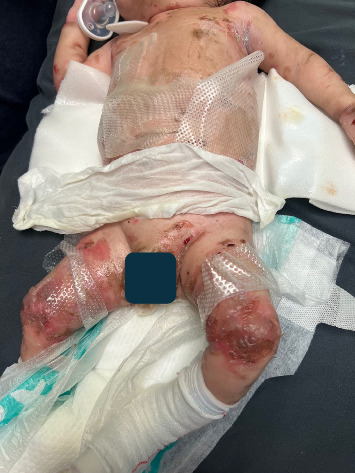
Patient with epidermolysis bullosa showing extensive ulcerations and blistering.

A 2-month-old male infant, weighing 3.72 kg, with EB simplex was scheduled for left inguinal hernia repair and circumcision (Figure 1). His medication history included daily morphine at 0.1 mg/kg for dressing changes. Given the clinical manifestations of EB simplex on his torso, extremities, and groin—while sparing the face—the family requested that airway manipulation be avoided if possible. After discussion of the risks and benefits, including potential failure necessitating conversion to general anesthesia, infection, bleeding, and nerve injury, the parents agreed to proceed.

The patient received premedication with intranasal dexmedetomidine (3 mcg/kg). A topical eutectic mixture of lidocaine and prilocaine cream (2.5% each) was applied to the lumbar spine area approximately 30 min prior to the procedure and covered with soft nonadhesive silicone foam dressing. Our institutional protocol for EB patients was strictly followed, which included the avoidance of adhesive for monitors, the utilization of lubricant for specific anesthesia equipment, and skin protective padding.

In our institution, IV access is routinely established after the spinal block in pediatric patients. However, in this patient, we elected to obtain intravenous access prior to block placement due to anticipated challenges in achieving access and to optimize duration of the spinal block for the surgical procedure itself. A well-lubricated mask was used for passive nitrous oxide administration, and a 22-gauge catheter was placed in the right forearm under ultrasound guidance. To facilitate IV securement and minimize movement, the patient received 5 mcg of fentanyl and 4 mcg of dexmedetomidine intravenously.

**Figure 2:**
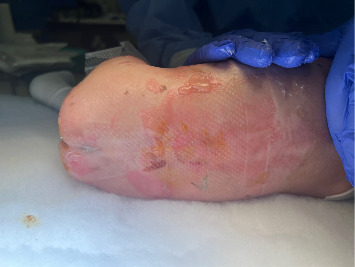
Patient positioned lateral decubitus prior to spinal block.

**Figure 3:**
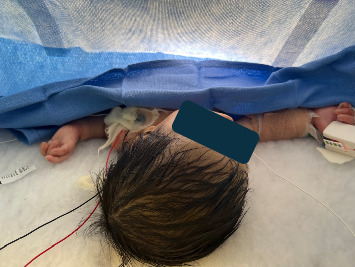
Patient asleep with natural airway during procedure done under spinal anesthesia.

The patient was positioned in lateral decubitus rather than the usual sitting position to minimize mechanical pressure caused by holding during spinal block placement (Figure 2). Using a Quincke 22G 1.5-inch spinal needle, a lumbar puncture was successfully performed at the L3-L4 level on the first attempt, followed by the injection of 0.5% bupivacaine (1 mg/kg) with 1 mcg/kg of clonidine and epinephrine wash [7]. This size spinal needle allows for quicker appreciation of cerebrospinal fluid reflux while not increasing the risk for postdural puncture headache in pediatric patients. The intercristal line can be used as an anatomical landmark for the L4-L5 lumbar spinal level, with the conus medullaris located between L1 and L2. Use of ultrasound to confirm anatomy can assist in correct level section [12]. The patient was then repositioned supine for the procedure (Figure 3). Assessing block height is challenging in this patient population to avoid unnecessary tissue trauma.

Eighty-seven minutes after spinal block, the patient began to move his legs and expressed mild discomfort. At this point, the hernia repair was completed, and circumcision was ongoing. The surgeon administered a penile block with 0.25% bupivacaine (1 mg/kg), and 2.5 mcg of IV fentanyl was given, allowing the patient to tolerate the remainder of the procedure without difficulty. Postoperative recovery was uneventful, and the patient was successfully discharged the same day.

## 3. Discussion

SA is a viable option for short urologic procedures in infants, especially when general anesthesia is contraindicated or best avoided. Infants receiving SA often fall asleep naturally due to sensory deafferentation, resulting in decreased arousal from reduced neural signaling and stimulation of the reticular activating system [13].

Our case is unique in that it involves the use of SA as the primary anesthetic technique for a procedure lasting over 90 min, which typically exceeds the standard duration of SA in infants when adjuvants like clonidine are used [14]. Although the motor block from SA using bupivacaine and clonidine typically lasts around 90 min, sensory block can persist longer and can be reinforced with additional local infiltration or nerve blocks, as well as supplemental sedatives and analgesics. These findings suggest that SA could be a viable option for a broader range of pediatric surgeries that were previously deemed unsuitable due to concerns about the limited duration of the block. Opioid adjuvants have also been considered but show less reliability for block duration extension and have potential for respiratory depression in neonates or preterm infants.

While previous cases have described the use of SA for EB patients, our case offers novel insights into this technique's application, particularly regarding the timing of IV access and patient positioning during neuraxial placement. Additionally, we highlight an updated approach to SA in infants, including premedication strategies to enhance block success rates. Caudal or epidural techniques as a sole anesthetic are restricted in pediatrics due to dosing limitation of local anesthetics to provide a dense enough surgical block.

Nasr et al. described the use of a combined spinal-epidural technique for a Mitrofanoff procedure lasting 150 min [10]. While this approach can extend surgical analgesia beyond the duration of the spinal block, it carries the risk of an incorrectly positioned epidural catheter, which could fail to deliver adequate analgesia and necessitate conversion to general anesthesia. Farber et al. reported the use of SA for open gastrostomy tube placement in a 12-week-old infant [11]. The spinal block was performed in the sitting position, and sedation was maintained during the case with propofol boluses and infusion. In contrast, we chose to place the spinal block in the lateral position, as achieving optimal positioning in the sitting position would have required significant manipulation and restraining of the infant. We also opted for dexmedetomidine instead of propofol, as it is more likely to preserve spontaneous ventilation and reduce the risk of airway obstruction.

Although airway management is challenging in this patient population, backup strategies need to be considered as rescue if spontaneous ventilation is impaired. The use of a well-lubricated laryngeal mask airway might be a less invasive approach that minimizes airway trauma compared to an endotracheal tube from tissue shear stress.

Pediatric institutions with established SA programs are well-positioned to offer this technique for patients when general anesthesia is contraindicated or undesirable. Our report offers novel insights into this technique's use in patients with EB and highlights an updated approach to SA in infants. Close communication with the surgical team is essential to accurately estimate procedure duration and ensure contingency plans are in place in case the procedure exceeds the anticipated time. SA offers numerous advantages and is feasible for managing EB patients, even for longer procedures. However, special precautions must be taken to optimize its success and safety in this unique patient population.

## Data Availability

Data sharing is not applicable to this article as no new data were created or analyzed in this study.
